# Excess weight, weight gain, and prostate cancer risk and prognosis: the PROCA-life study

**DOI:** 10.2340/1651-226X.2024.32953

**Published:** 2024-04-09

**Authors:** Martin Støyten, Tore Knutsen, Einar Stikbakke, Ingvild Agledahl, Tom Wilsgaard, Anne Elise Eggen, Elin Richardsen, Edward Giovannucci, Inger Thune, Hege S. Haugnes

**Affiliations:** a Institute of Clinical Medicine, UIT – The Arctic University, Tromsø, Norway; b Department of Urology, University Hospital of North Norway, Tromsø, Norway; c Department of Oncology, University Hospital of North Norway, Tromsø, Norway; d Institute of Community Medicine, UIT-The Arctic University, Tromsø, Norway; e Department of Pathology, University Hospital of North Norway, Tromsø, Norway; f Department of Medical Biology, UIT – The Arctic University, Tromsø, Norway; g Departments of Nutrition and Epidemiology, Harvard T.H. Chan School of Public Health, Boston, MA, USA; h Insitute of Clinical Medicine, University of Oslo, Oslo, Norway; i Department of Oncology, Oslo University Hospital, Norway

**Keywords:** Prostate cancer, incidence, body mass index, weight change, mortality

## Abstract

**Background:**

Studies of excess weight and weight changes throughout adult life for prostate cancer (PCa) risk and prognosis have shown inconsistent results.

**Methods:**

In a population-based cohort, the Prostate Cancer Study throughout life (PROCA-*life*), 16,960 healthy men from the prospective cohort Tromsø Study (1994–2016) were included. Body mass index (BMI) and weight were measured at all four attendings, and weight change was calculated as the difference between the first and last of either Tromsø4, Tromsø5 or Tromsø6. Overall, 904 men developed PCa during 16 years of follow-up, and Poisson regression with fractional polynomials was used to investigate trends in incidence. Cox proportional hazard and logistic regression models were used to study associations between measurements of BMI and weight change and PCa risk, severity, and mortality.

**Results:**

At study entry, 46% of the participants (median age 44 years) were overweight, and 14% were obese (BMI > 30 kg/m^2^). We observed a 127% increase in overall age adjusted PCa incidence in the cohort during 1995 through 2019. No overall associations between BMI or weight change and PCa risk were observed. However, in sub-group analysis, weight gain among obese men was associated with a three-fold higher PCa risk (HR 3.03, 95% CI 1.39–6.58) compared with obese men with stable weight. Overweight was associated with lower risk of metastatic cancer (OR 0.48, 95% CI 0.30–0.75) at diagnosis. Men with obesity had higher risk of PCa-specific death (HR 1.72, 95% CI 1.03–2.88), while nonsmoking obese PCa cases had two times higher PCa-specific mortality compared with normal weighted PCa cases (HR 2.10, 95% CI 1.11–3.70).

**Interpretation:**

In our cohort, weight gain among obese men was associated with higher risk of PCa, and obesity was associated with higher PCa-specific mortality, especially among nonsmokers. The relationship between weight and risk for PCa remains complicated, and future studies are needed to determine clinical implications.

## Background

The global increase in overweight and obesity parallels the increase in prostate cancer (PCa) [1–4], one of the most common cancer types among men worldwide [5]. Thus, any potential modifiable risk factor that may reduce the burden of PCa is important to study in detail across populations and during lifetime.

Several studies have supported a positive association between obesity and PCa development and mortality [6, 7], in contrast to others [8, 9]. A recent meta-analysis reported that a higher body mass index (BMI) was associated with a higher overall and PCa-specific mortality [10]. Other studies have reported a positive association between obesity and aggressive PCa [11, 12], but some studies have found a positive association, only among nonsmokers, between long-term weight gain and fatal PCa [13, 14]. Moreover, a lower risk for fatal PCa with increasing BMI at age 18 was suggested, while a higher BMI later in life was associated with higher risk of PCa [14, 15]. These observations are also in part supported by others [8, 9, 16]. In contrast, meta-analyses failed to detect any association between high BMI and obesity and PCa incidence [11, 17, 18].

Several biological mechanisms have been hypothesized to explain the variation observed between excess weight and PCa risk and mortality. Excess weight at a young age may delay maturation of the prostate and lower testosterone levels [19] and prevent development of PCa among the youngest men at risk. In contrast, excess weight and adipose tissue locally in the prostate and periprostatic tissue may result in more chronic inflammation [15], increased angiogenesis [20] and secretion of cytokines [21], stimulating PCa development later in life.

Furthermore, excess weight has been associated with lower levels of prostate-specific antigen (PSA) [18]. These findings may in part be explained by higher plasma volume and hemodilution [22]. Moreover, the geographical differences in PCa screening with PSA have been shown to correlate strongly with the observed differences in PCa incidence [23, 24], but differences in screening rates cannot fully explain the variation in PCa incidence, since these differences were observed before PSA-testing became available [24].

To our knowledge, most previous studies investigating the association between excess weight and PCa incidence, aggressiveness, and mortality include only a single measure of self-reported weight and BMI. However, weight gain assessed by repeated weight measurements was associated with higher PCa risk [25], but in a large meta-analysis, there was no association between adult weight gain and PCa [26]. Since obesity is a possible modifiable lifestyle factor, it is essential to improve the understanding of how excess weight and weight change in adult life may influence PCa development.

Thus, the aim of the present study was to evaluate the changes in PCa incidence throughout the last three decades. In addition, we wanted to explore whether adult excess weight and changes in prediagnostic weight are linked to PCa risk, severity, and mortality in a large Norwegian population-based cohort study with high repeated attendance rates including multiple measurements of height and weight.

## Material and methods

In the Prostate Cancer Throughout Life Study (PROCA-*life* study), 17,542 men aged ≥ 25 years at entry, who participated in the Tromsø Study surveys, 1994–1995 (Tromsø4), 2001 (Tromsø5), 2007–2008 (Tromsø6), or 2015–2016 (Tromsø7) were included. Men who were diagnosed with any cancer prior to attending the study, and men who developed any cancer during the first year after study entry were excluded to account for the possibility that undiagnosed cancer or severe illness could affect the results (*n* = 555). Men with missing measurements of height and weight at first study entry were also excluded (*n* = 27). A total of 16,960 men were included in the final study population, of whom 904 developed incident PCa during the follow-up period (December 31, 2019). Weight change analyses were performed on 5,680 men, of whom 459 developed PCa during follow-up (Supplementary Figure).

Personal invitations were sent to all age-eligible men, and nonresponders were given one reminder. The procedures of invitations, screening, and examinations were almost identical in the four included surveys [27, 28]. The attendance rates in the surveys varied between 66% and 75% [27]. All participants completed questionnaires, provided biological specimens, and underwent measurements and clinical examinations at each survey.

### Questionnaires

Questionnaires sent out by invitation, were filled in at home and brought to the study site where they were checked for completeness and consistency. The questionnaires included items among others chronic diseases, socioeconomic-and lifestyle factors [28].

### Assessments of anthropometric measurements and serum samples

Height and weight were measured at each of the four surveys (1994–1995, 2001, 2007/2008, 2015/2016) with the participants wearing light clothes and no shoes. Height was measured to the nearest centimeter (cm) and weight to the nearest kilogram (kg) using an electronic scale. BMI was calculated using the formula; weight (kg)/height^2^ (m^2^) [28, 29]. PSA measurements were performed on cancer cases only, as part of clinical routine in diagnosis and follow-up (1990–1994 Stratus® PSA Fluorometric Enzyme Immunoessay, 1994–2001 AxSYM Psa Reagent Pack, Abbot®, 2001 Bayer^®^ PSA Reagens Pack Immuno I (Prod. Nr. T01-3450-51, Technicon Immuno I).

### Identification of PCa cases, PCa characteristics and medical charts during follow-up

All PCa cases were identified through linkage to the Cancer Registry of Norway by using the unique national 11-digit identification number. Information on emigration and main cause of death was obtained from the National Population Registry of Norway and the Cause of Death Registry at the Norwegian Institute of Public Health, respectively.

Histopathological information for the PCa cases were obtained from histopathological records, and all histopathological specimens were reexamined by the same experienced uro-pathologist (ER) and reclassified according to the latest International Society of Urological Pathology (ISUP) guidelines on Gleason Score and ISUP Grade Group (ISUP GG) [30]. The TNM classification was based on status in the medical records, according to the 7th edition of the Union for International Cancer Control (UICC) TNM classification system [31]. To avoid the T-stage migration introduced by gradual introduction of MRI in PCa diagnosis in the period, we recorded T-stage solely based on digital rectal examination, as in the following 8th version of UICC TNM classification.

Risk group categorization was made according to a modification of the D’Amigo classification [32]. Low risk was defined as ISUP GG 1, PSA < 10 and T-stage ≤ cT2A; intermediate risk as ISUP GG 2/3 or PSA 10–20 or T-stage cT2b-cT2c; high risk as ISUP GG 4 or 5, or PSA > 20, or T-stage ≥ cT3a.

Medical records for PCa cases were reviewed to obtain detailed clinical data by trained physicians (MS, TK and ES). Follow-up time was calculated from the date of study entry to the date of death, date of PCa, date of a different cancer, emigration, or the end of follow-up (December 31, 2019), whichever came first.

### Statistical analysis

Descriptive statistics were presented as mean with standard deviation (SD), median with interquartile range (IQR), or percent with numbers. Trends in incidence for PCa during follow-up, as new PCa cases per 1,000 person-years at risk, were calculated using Poisson regression with fractional polynomials of calendar year as the main predictor and age as covariate. The best -fitting fractional polynomials was chosen using the Akaike information criterion.

Cox proportional hazard regression models were used to study associations between incident PCa and overall and PCa-specific mortality as dependent variables and measurements of BMI and weight change as independent variables. Logistic regression models were used to study associations between BMI, weight change, and severe PCa, using risk categorization (high risk nonmetastatic or metastatic) and high PSA (> 20) as binary dependent variables in separate models and most recent BMI and weight change before diagnosis as independent variables, adjusted for age at diagnosis. In separate models, BMI was included as a continuous variable and as a categorical variable with cut offs < 25.0 kg/m^2^ (normal weight), ≥ 25 kg/m^2^–< 30.0 kg/m^2^ (overweight), ≥30 kg/ m^2^ (obesity), using < 25.0 kg/m^2^ as reference level.

Sub-groups of the cohort with two or more weight measures were included in models assessing weight change as a risk factor for incident and aggressive PCa and mortality. Weight gain or loss was calculated as relative weight change in percentage of entry weight between Tromsø4 and 5, between Tromsø4 and 6, or between Tromsø5 and 6, depending on which study they attended, using the formula: Most recent weight - entry weight/entry weight* 100. For those who had participated in all three surveys, we defined the weight change from weight at entry to the most recent weight measure: weight change between Tromsø4 and 6. Weight change was categorized as stable (less than ± 3% change in weight at entry), small increase (≥ 3%, but < 5%), large increase (≥ 5%), small loss (≤–3%, but >–5%), or large loss (≤–5%) in accordance with recommendations and previous studies [33]. The stable group was used as reference level in all models.

To study the association between variation in weight change and BMI, and PCa development in more detail, we performed subgroup analyses split by age at entry (tertiles) and BMI at entry (< 25.0 kg/m^2^, ≥ 25 kg/m^2^ – <30.0 kg/m^2^, ≥ 30 kg/ m^2^). We also performed analyses separately among smokers and nonsmokers to evaluate the possible effect of smoking.

Based on suggested biological mechanisms influencing PCa development and prognosis, the following variables were tested and included when appropriate as potential confounders: smoking at entry (categorical), physical activity at entry (categorical), alcohol consumption at entry (categorical), and highest level of education (categorical).

To account for the impact of age, we used age as time scale in the Cox models. The proportional hazard assumption was investigated graphically by assessing log minus log survival curves, and the PH assumption was met in all analyses. Survival curves of all cause and PCa-specific mortality were presented by BMI in groups and by weight change using stratified Cox models adjusted for smoking, physical activity, education, alcohol, and age at diagnosis, using date as timescale.

All statistical tests were two sided using a significance level of 5%. Statistical analyses were conducted with STATA, version 17 (StataCorp. 2021. Stata: Release 17. Statistical Software. College Station, TX: StataCorp LLC).

## Results

### Characteristics and PCa incidence

Among the 16,960 men included with a median age at entry of 44 years and a mean BMI at entry of 26 kg/m^2^, 46% of these men were overweight and 14% obese. Among the 5,680 men with repeated measurements of height and weight, 43% gained more than 5% of body weight (kg).

A total of 904 men developed PCa, during the median follow-up time of 16 years, with a median age at diagnosis of 68 years. The median PSA level at diagnosis was 9.8 mg/L. The proportion of PCa cases with high-grade cancer (ISUP GG ≥ 4) was 19%, and 26% of the PCa cases had high risk disease and 12% had metastatic disease at diagnosis. Among the PCa cases, 311 (34%) men died during follow-up of whom 154 (17%) were PCa-specific deaths (Table 1).

**Table 1:** Distribution of characteristics of men (overall, noncases, and cases) at study entry and characteristics of PCa cases. The PROCA life study.

Characteristic	Overall (n = 16,960)	Noncases (n = 16,056)	PCa cases (n = 904)
Age at entry, year, median (IQR)	44 (37–54)	44 (37–54)	52 (46–60)
Observation time, year, median (IQR)^a^	21 (7–25)	23 (7–25)	16 (10–20)
**Education**			
Elementary school	3,287 (19)	3,045 (19)	242 (27)
High school	3,875 (23)	3,667 (23)	208 (23)
< 4 years at college/university	2,493 (15)	2,371 (15)	122 (14)
≥ 4 years at college/university	7,305 (43)	6,973 (44)	332 (37)
**Clinical variables, mean (SD)**			
Height, cm	178 (7)	178 (7)	177 (6)
Weight, kg	81 (13)	81 (13)	80.0 (12)
BMI, kg/m^2^	26 (4)	26 (4)	26 (3)
**BMI categories**			
Normal weight (< 25 kg/m^2^)	6,828 (40)	6,451 (40)	377 (42)
Overweight (≥ 25 & < 30 kg/m^2^)	7,800 (46)	7,377 (46)	423 (47)
Obesity (≥ 30 kg/m^2^)	2,332 (14)	2,228 (14)	104 (12)
**Weight change, categories**			
≤–5%	626 (11)	572 (11)	54 (12)
≤–3% to >–5%	327 (5)	294 (6)	33 (7)
< 3% to >–3%	1,636 (29)	1,495 (29)	141 (31)
≥ 3% to < 5%	633 (11)	574 (11)	59 (13)
≥ 5%	2,458 (43)	2,286 (44)	172 (38)
**Blood pressure (BP), mean (SD)**			
Systolic BP, mmHg	135 (17)	135 (17)	140 (19)
Diastolic BP, mmHg	79 (11)	79 (11)	83 (12)
**Lifestyle variables**			
Current smokers	5,506 (33)	5,225 (33)	281 (31)
Alcohol use
Teetotaler	1,307 (9)	1,234 (8)	73 (8)
0–4 times per month	11,316 (67)	10,745 (68)	571 (64)
> 4 times per month	4,187 (25)	3,933 (25)	254 (28)
**Physical activity**			
Sedentary	2,949 (18)	2,808 (18)	141 (16)
Moderate	10,474 (62)	9,839 (62)	635 (71)
High	3,405 (20)	3,285 (21)	120 (13)
**Characteristics among PCa cases**			
Age at diagnosis, year, median (IQR)			68 (63–74)
PSA at diagnosis, (µg/L), median (IQR)			9.8 (6.5–19.1)
**ISUP grade group^b^**			
1–3 (Gleason score 6–7)			633 (81)
4–5 (Gleason score 8–10)			151 (19)
**cT stage at diagnosis^c^**			
T1–T2			604 (75)
T3–T4			174 (21)
**Risk group^d^**			
Low			148 (17)
Intermediate			337 (38)
High			227 (26)
Metastatic			108 (12)
**Mortality**			
Overall mortality	3,533 (21)	3,222 (20)	311 (34)
PCa-specific mortality	154 (1)	0 (0)	154 (17)
CVD-specific death	1,115 (7)	1,072 (7)	43 (5)
Other cause of death	2,247 (13)	2,133 (13)	114 (13)

Data are presented as numbers (%) unless otherwise stated.

PCa: prostate cancer; n: numbers; IQR: inter quartile range; SD, standard deviation; BMI: body mass index; BP: blood pressure; PSA: prostate-specific antigen; ISUP-score: International society of urological pathology-score; CVD: cardiovascular disease; SD: standard deviation.

a Time from first attendance to either end of follow-up (December 31, 2019), PCa, death or emigration.

b ISUP grade group [30].

c clinical T-stage at diagnosis assessed by digital rectal exploration.

d Risk groups are defined according to D-Amico: Low risk was defined as ISUP GG 1, PSA < 10 and T-stage ≤ cT2A; intermediate risk as ISUP GG 2/3 or PSA 10–20 or T-stage cT2b-cT2c; high risk as ISUP GG 4 or 5, or PSA > 20, or T-stage ≥ cT3a [32].

**Figure 1:**
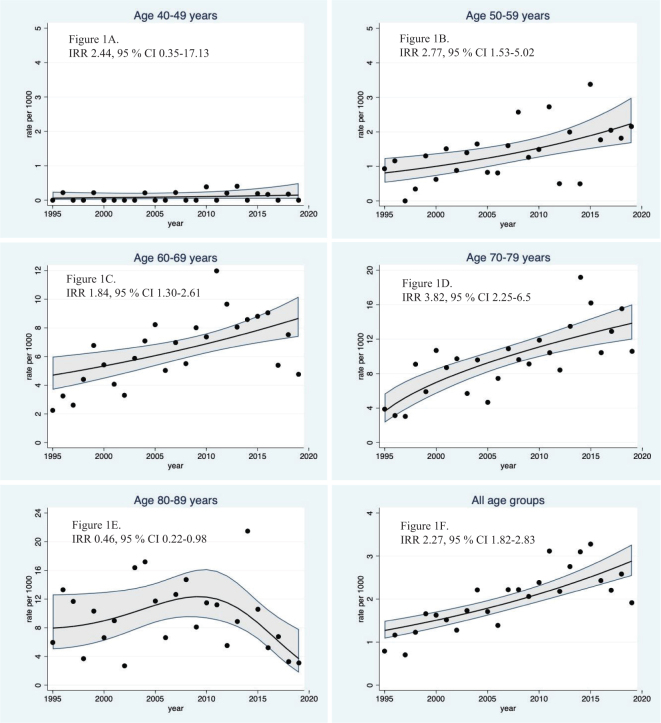
Age-adjusted time trends in incidence rates of prostate cancer according to age at diagnosis for men in the PROCA life Study during 1995–2019. Dots represent annual incidence rate pr 1,000 person-years, with solid lines representing best fitted regression line for trend, with 95% confidence intervals. Y-axis scale differ by age groups. Test of trends are significant for all groups except for the age-group 40–49 (Figure 1A-F). IRR, incidence rate ratio, compares year 2019 and 1995.

We observed an annual increase in PCa incidence for all age groups, except for the age group 80–89 years. Furthermore, we observed a 127% increase in overall age-adjusted PCa incidence in the whole cohort through the period from 1995 to 2019 (incidence rate ratio of 2.27; 95% CI 1.82–2.83) (Figure 1).

### BMI, weight change, and PCa risk and severity

Overall, we observed no associations between BMI or weight change and incident PCa (Supplementary Table). In subgroup analysis, men with a BMI ≥ 30kg/m^2^ at study entry, and a weight gain ≥ 5% during follow-up, had a three-fold higher risk for incident PCa compared with obese men with stable weight (HR 3.03, 95% CI 1.39–6.58) (Table 2).

**Table 2:** Hazard ratios (HRs) for incident prostate cancer according to prediagnostic weight change stratified by prediagnostic, baseline body mass index (BMI). The PROCA life Study.

		BMI < 25 kg/m^2^ Total N = 2,378			BMI 25 – 30 kg/m^2^ Total N = 2,702			BMI ≥ 30 kg/m^2^ Total N = 600	
		Model 1^a^ HR (95% CI)	Model 2^b^ HR (95% CI)		Model 1^a^ HR (95% CI)	Model 2^b^ HR (95% CI)		Model 1^a^ HR (95% CI)	Model 2^b^ HR (95% CI)
Weight change	N=^c^			N=^c^			N=^c^		
≤–5%	15	0.84 (0.48–1.48)	0.83 (0.47–1.47)	27	1.09 (0.70–1.69)	1.10 (0.70–1.72)	12	1.87 (0.78–4.45)	1.75 (0.72–4.24)
>–5% to ≤–3%	16	1.32 (0.76–2.30)	1.23 (0.70–2.18)	14	0.96 (0.54–1.70)	0.95 (0.54–1.69)	3	2.28 (0.61–8.46)	2.55 (0.68–9.51)
>–3% to < 3%	60	1 (reference)	1 (reference)	72	1 (reference)	1 (reference)	9	1 (reference)	1 (reference)
≥ 3% to < 5%	24	1.19 (0.74–1.91)	1.18 (0.73–1.90)	26	0.92 (0.59–1.44)	0.92 (0.60–1.45)	9	**3.12 (1.23–7.94)**	**3.53 (1.38–9.03)**
≥ 5%	72	0.94 (0.66–1.33)	0.91 (0.64–1.29)	73	0.93 (0.67–1.30)	0.96 (0.69–1.34)	27	**2.96 (1.37–6.41)**	**3.03 (1.39–6.58)**
P trend		0.861	0.807		0.511	0.592		0.085	0.057

a Adjusted for age.

b Adjusted for smoking, physical activity, education level, and alcohol at the same time of baseline.

c Number of incident prostate cancer cases

Among smokers who were overweight at study entry (BMI 25–30 kg/m^2^), we observed a lower risk of PCa (HR 0.75, 95% CI 0.59–0.97). There were no associations between overweight, obesity, or weight gain and incident PCa among nonsmokers (data not presented).

Men who were overweight had a lower risk of metastatic disease at the time of diagnosis compared with normal weighted men (OR 0.48, 95% CI 0.30–0.75) (Table 3). However, there was no association between overweight and high-risk nonmetastatic PCa. Obesity (BMI ≥ 30 kg/m^2^) or weight change was not associated with high-risk or metastatic PCa. When comparing men who were overweight with normal weighted men, we observed a lower risk of high PSA (OR 0.69, 95% CI 0.48–0.99). Obesity (BMI ≥ 30 kg/m^2^) or weight change were not associated with high PSA (data not shown).

**Table 3:** Age-adjusted odds ratios (OR) for high risk, nonmetastatic, and metastatic prostate cancer according to pre-diagnostic body composition (BMI and weight change). The PROCA *life* Study.

	High risk, nonmetastatic PCa^a^		Metastatic PCa^b^	
	OR (95 % CI)		OR (95 % CI)	
BMI, kg/m^2^	N=^c^		N=^c^	
< 25	78/281	1 (reference)	51/281	1 (reference)
25 to < 30	111/411	0.97 (0.69–1.36)	40/411	**0.48 (0.30–0.75)**
≥ 30	37/127	1.06 (0.66–1.69)	17/127	0.68 (0.37–1.25)
**BMI, continuous**				
Per SD^d^		1.04 (0.88–1.23)		**0.79 (0.62–1.00)**
**Weight change**	N=^c^		N=^c^	
≤–5%	13/53	0.82 (0.39–1.70)	10/53	0.84 (0.35–2.02)
>–5% to ≤–3%	13/31	1.86 (0.83–4.17)	5/31	0.79 (0.26–2.42)
>–3% to < 3%	37/135	1 (reference)	23/135	1 (reference)
≥ 3% to < 5%	20/57	1.45 (0.74–2.82)	6/57	0.52 (0.19–1.45)
≥ 5%	33/159	0.73 (0.43–1.26)	15/159	0.65 (0.31–1.37)

BMI: Body Mass Index; ISUP: International society of urological pathology; N: numbers; SD: standard deviation. BMI measured as close to the time of diagnosis as possible.

a High risk, nonmetastatic defined as ISUP grade group 4 or 5, or PSA >20, or cT-stage ≥ T3a, or N1, and M0.

b Metastatic defined as M1 or PSA>100

c Number of cases with the given disease characteristics / Number of total cases in the group

d Standard deviation for BMI was calculated to 4 kg/m^2^

### BMI, weight gain/change, and mortality

**Figure 2:**
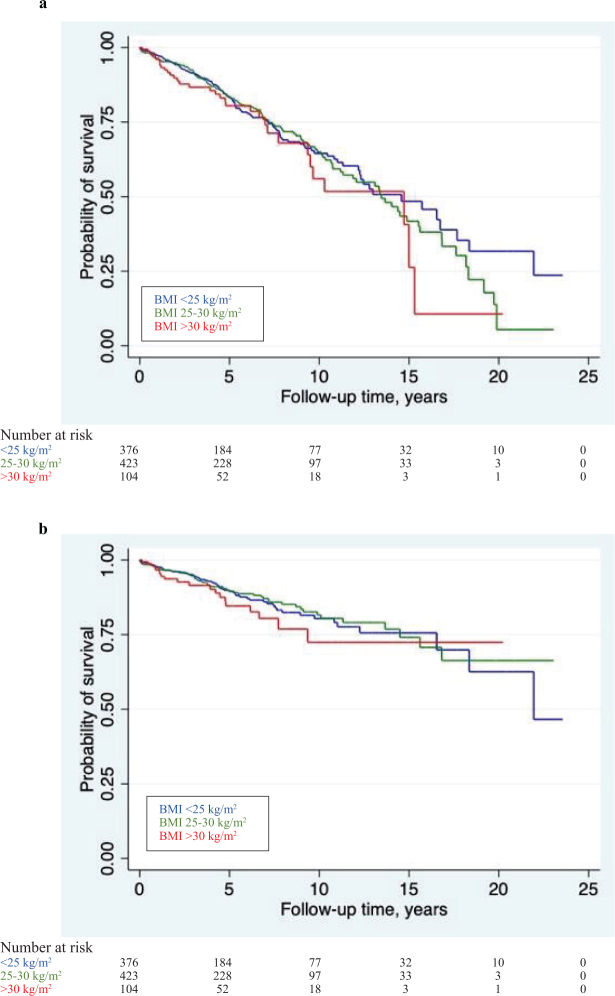
Survival curves for men diagnosed with prostate cancer in the PROCA life study during 1995-2019, stratified by body mass index (BMI) (a) overall mortality, (b) prostate-cancer mortality.

Obese men (BMI ≥ 30 kg/m^2^) had an increased PCa-specific mortality compared with normal weighted men (HR 1.72, 95% CI 1.03–2.88) (Table 4, Figure 2). However, there were lower risks of both overall mortality and PCa-specific mortality with large weight gain (p for trends 0.005 and 0.003, respectively). Men with weight gain of more than 5% had lower overall mortality (HR 0.59, 95% CI 0.39–0.91) and lower PCa-specific mortality (HR 0.52, 95% CI 0.28–0.95) compared with men with stable weight.

**Table 4:** Hazard ratios (HRs) for overall and prostate cancer (PCa) specific mortality for PCa cases according to BMI at baseline. Data are presented for the overall PCa population, and stratified for nonsmokers and smokers at baseline. The PROCA *life* study.

		Overall mortality			PCa Specific mortality		
		Overall HR (95% CI)	Nonsmokers HR (95% CI)	Smokers HR (95% CI)		Overall HR (95% CI)	Nonsmokers HR (95% CI)	Smokers HR (95% CI)
BMI group (kg/m^2^)	N=^a^				N=^a^			
< 25.0	130/377	1 (reference)	1 (reference)	1 (reference)	66/377	1 (reference)	1 (reference)	1 (reference)
25.0 to < 30	145/423	1.09 (0.85–1.39)	1.31 (0.95–1.80)	0.81 (0.55–1.21)	67/423	0.98 (0.69–1.40)	1.08 (0.70–1.68)	0.76 (0.41–1.42)
≥ 30	36/104	1.44 (0.98–2.12)	**1.79 (1.10–2.89)**	0.98 (0.50–1.95)	21/104	**1.72 (1.03–2.88)**	**2.02 (1.11–3.70)**	1.03 (0.35–3.02)
P trend		0.097	**0.014**	0.539		0.160	0.066	0.627
**BMI, continuous**								
per SD^b^		1.14 (0.99–1.31)	**1.33 (1.12–1.57)**	0.86 (0.68–1.08)		1.17 (0.97–1.41)	**1.29 (1.03–1.63)**	0.93 (0.65–1.34)

All analyses are adjusted for physical activity, education level, and alcohol at the time of baseline. Analyses in overall population are adjusted for smoking status at baseline.

a Number of deaths/Number of total cases in the group.

b Standard deviation for BMI was calculated to 4 kg/m^2^.

Among nonsmokers, obesity was associated with higher overall and PCa-specific mortality (HR 1.79, 95% CI 1.10–2.89 and HR 2.02, 95% CI 1.11–3.70, respectively) (Table 4). Neither overall nor PCa-specific mortality was increased among smokers with obesity. We observed no associations between weight gain and overall or PCa-specific mortality among nonsmokers (data not shown), while smokers with a weight gain > 5% had lower overall mortality and PCa-specific mortality compared with smokers with stable weight (HR 0.28, 95% CI 0.12–0.63, and HR 0.16, 95% CI 0.04–0.60, data not presented in table).

## Discussion

In our prospective cohort study, we observed a 127% increase in PCa incidence during 16 years of follow-up. We observed no overall association between BMI and PCa risk, but importantly weight gain among men who were obese at study entry was associated with a three-fold higher risk for incident PCa compared with obese men with stable weight. Moreover, obese men at study entry had a 70% higher PCa-specific mortality compared with normal weighted men, while nonsmoking obese men diagnosed with PCa had a two times higher PCa-specific mortality compared with normal weighted nonsmoking men.

The strong increase in age-adjusted incidence of PCa in our cohort follows the well-known pattern for high-income countries, and Norway has together with the other Nordic countries, the highest incidence rate of PCa in Europe [7, 34, 35]. This increase in incidence also parallels the increase in excess weight in the Norwegian population as the prevalence of obesity in adult men has increased substantially during the study period, from 10% in 1994–95% to 21% in 2007–2008 [4]. In addition, the increased incidence parallels in time with a widespread opportunistic use of PSA-tests, and although Norway has never had an organized PSA screening program [36], opportunistic use of PSA testing has been shown to increase with age [37].

The positive association between weight gain and incident PCa in obese men in our study is supported by Wang and colleagues who observed a 3.7-fold higher PCa risk among obese men with weight gain [25]. One possible explanation of these findings could be that obese men visit their doctor more often due to other health problems than normal weighted men and are therefore more likely to have their PSA measured resulting in a higher likelihood of having PCa diagnosed.

The lack of an overall association between BMI and PCa risk as reported herein are supported by some [11, 17, 18] but are in contrast to others [16, 38–40]. A possible explanation for the lack of association in our study may be hemodilution of PSA and/or increased size of the prostate with lower probability of finding a malignant focus on random biopsies [12, 39]. Interestingly, previous meta-analyses have found a positive association between obesity and incidence of PCa observed in the leaner populations in Europe and Australia and no association in the potentially more obese US population and suggested that different patterns of PSA screening may be the cause [39, 40]. Another explanation may be that the association between excess weight and PCa development may vary according to the time period throughout life where exposure to excess weight may occur [14, 41, 42]. A high BMI at a young age was observed to be negatively associated with PCa, while weight gain in adult life may increase the risk for incident PCa [9, 14, 15, 20]. Our cohort may therefore include the overweight men who received the suggested protective benefits of adiposity at a younger age before study entry and fewer men who have their weight gain later in adult life. This may also explain the inverse association between overweight and risk for metastatic PCa observed in our study, which is in contrast to other studies [17, 18, 38]. However, previous studies on the association between BMI and metastatic PCa is limited, and this topic remains controversial.

There are several biological mechanisms that support a variation in the association between excess weight during lifetime and prostate development [19, 20, 42]. Androgens play a major role in the development and maturation of the normal prostate as well as for risk of PCa development [43], and androgens are lower in obese men [44]. Recently, in a study based on data from a UK biobank using Mendelian randomization, they observed that increased bioavailable testosterone was associated with higher risk of PCa. They also observed that lower bioavailable testosterone was associated with higher BMI [42]. On the other hand, the increased aromatase activity in obese men leading to higher concentrations of estradiol may promote PCa development [45]. Furthermore, adipose tissue including peri-prostatic adipose tissue may exert both systemic and local hormonal effects through secretion of adipokines and cytokines that may stimulate PCa development [21].

We observed an inverse association in the total study population between overweight and high PSA, in line with a recent study showing an inverse association between PSA and BMI [18]. The lower levels of observed PSA in overweight men compared with normal-weight men may also account for some of the observed inverse association between overweight and metastatic PCa. On the other hand, obesity and metabolic syndrome have been associated with lower urinary tract symptoms (LUTS) [46, 47], which may entail a more widespread use of PSA-testing, thus increasing the probability of being diagnosed with PCa at an earlier stage. Even so, the degree of LUTS have not been found to correlate with PCa [48, 49].

The higher risk of PCa-specific mortality among those who were obese at study entry is supported [10]. Interestingly, in our study, the higher risk of all fatal events in PCa was only observed among nonsmokers and not in smokers and support findings in the National Institutes of Health-American Association of Retired Persons (NIH-AARP) Diet and Health Study [14]. They observed that smoking status modified the relationship between BMI and fatal PCa. Additionally, obesity may also be a risk factor for complications during surgery, and side effects from oncological treatment [50]. Furthermore, our study observed a lower risk of overall mortality among PCa cases with weight gain, which is in part supported by findings in the same study population who found that men with a weight loss had higher all-cause mortality [51].

The present study has some major strengths that include a population-based cohort study with high attendance rate and anthropometric measurements that lessen the chance of biased observations. The high completeness rates of identification of PCa cases (Cancer Registry of Norway) and identification of death and emigration (Cause of Death Registry) have been estimated to be very close to complete, 99% [52]. All medical records (clinical and histological) for the PCa patients were carefully reviewed. We limited our study population to men who had no history of cancer before or within the first year after study inclusion to lessen the chance that previous cancer could influence our results.

Our study also has some weaknesses. The prostate carcinogenesis entails a protracted course that can initiate as early as in the third decade of life, and thus a young cohort as the present may not have the ability to fully explore the effect of weight gain on PCa risk during adult life. Moreover, the high 10-year survival rate from PCa together with relatively short follow-up time after diagnosis limits the evaluation of overall and PCa-specific mortality. A longer prediagnostic and postdiagnostic follow-up period including changes in weight would have strengthened our findings.

## Interpretation

In our cohort, weight gain among obese men was associated with higher risk of PCa, and obesity was associated with higher PCa-specific mortality, especially among nonsmokers.

These findings support that excess weight may be a potential important modifiable factor associated with PCa, but the relationship observed between excess weight during adult life and fatal PCa is complex and age at exposure and smoking habits may interact. Further research is needed to understand the role of excess weight in clinical settings to prevent PCa and improve PCa survival.

## Supplementary Material

Excess weight, weight gain, and prostate cancer risk and prognosis: the PROCA-life study

Excess weight, weight gain, and prostate cancer risk and prognosis: the PROCA-life study

## Data Availability

The data are not available due to restrictions from the data protection officer and the Regional Committee for Medical and Research Ethics.

## References

[1] Finucane MM, Stevens GA, Cowan MJ, et al. National, regional, and global trends in body-mass index since 1980: systematic analysis of health examination surveys and epidemiological studies with 960 country-years and 91 million participants. Lancet. 2011;377(9765):557–67. 10.1016/S0140-6736(10)62037-5 21295846 PMC4472365

[2] Meyer HE, Tverdal A. Development of body weight in the Norwegian population. Prostaglandins Leukot Essent Fatty Acids. 2005;73(1): 3–7. 10.1016/j.plefa.2005.04.003 15916890

[3] Midthjell K, Lee CM, Langhammer A, et al. Trends in overweight and obesity over 22 years in a large adult population: the HUNT Study, Norway. Clin Obes. 2013;3(1–2):12–20. 10.1111/cob.12009 23935708 PMC3734732

[4] Jacobsen BK, Aars NA. Changes in body mass index and the prevalence of obesity during 1994–2008: repeated cross-sectional surveys and longitudinal analyses. The Tromsø Study. BMJ Open. 2015;5(6):e007859. 10.1136/bmjopen-2015-007859 PMC4466626 26070799

[5] Sung H, Ferlay J, Siegel RL, et al. Global cancer statistics 2020: GLOBOCAN estimates of incidence and mortality worldwide for 36 cancers in 185 countries. CA Cancer J Clin. 2021;71(3):209–49. 10.3322/caac.21660 33538338

[6] Wilson RL, Taaffe DR, Newton RU, Hart NH, Lyons-Wall P, Galvão DA. Obesity and prostate cancer: a narrative review. Crit Rev Oncol Hematol. 2022;169:103543. 10.1016/j.critrevonc.2021.103543 34808374

[7] Gandaglia G, Leni R, Bray F, et al. Epidemiology and prevention of prostate cancer. Eur Urol Oncol. 2021;4(6):877–92. 10.1016/j.euo.2021.09.006 34716119

[8] Kazmi N, Haycock P, Tsilidis K, Lynch BM, Truong T. Appraising causal relationships of dietary, nutritional and physical-activity exposures with overall and aggressive prostate cancer: two-sample Mendelian-randomization study based on 79148 prostate-cancer cases and 61106 controls. Int J Epidemiol. 2020;49(2):587–96. 10.1093/ije/dyz235 31802111

[9] Onerup A, Mehlig K, Af Geijerstam A, et al. Associations between BMI in youth and site-specific cancer in men – a cohort study with register linkage. Obesity. 2023;32(2):376–89. 10.1002/oby.23942 37927128

[10] Rivera-Izquierdo M, Pérez de Rojas J, Martínez-Ruiz V, et al. Obesity as a risk factor for prostate cancer mortality: a systematic review and dose-response meta-analysis of 280,199 patients. Cancers. 2021;13(16):4169. 10.3390/cancers13164169 34439328 PMC8392042

[11] Zhang X, Zhou G, Sun B, et al. Impact of obesity upon prostate cancer-associated mortality: a meta-analysis of 17 cohort studies. Oncol Lett. 2015;9(3):1307–12. 10.3892/ol.2014.2841 25663903 PMC4315023

[12] Allott EH, Masko EM, Freedland SJ. Obesity and prostate cancer: weighing the evidence. Eur Urol. 2013;63(5):800–9. 10.1016/j.eururo.2012.11.013 23219374 PMC3597763

[13] Dickerman BA, Ahearn TU, Giovannucci E, et al. Weight change, obesity and risk of prostate cancer progression among men with clinically localized prostate cancer. Int J Cancer. 2017;141(5):933–44. 10.1002/ijc.30803 28543830 PMC5518616

[14] Kelly SP, Lennon H, Sperrin M, et al. Body mass index trajectories across adulthood and smoking in relation to prostate cancer risks: the NIH-AARP Diet and Health Study. Int J Epidemiol. 2019;48(2): 464–73. 10.1093/ije/dyy219 30376043 PMC6469294

[15] Genkinger J, Wu K, Wang M, et al. Measures of body fatness and height in early and mid-to-late adulthood and prostate cancer: risk and mortality in The Pooling Project of Prospective Studies of Diet and Cancer. Ann Oncol. 2020;31(1):103–114. 10.1016/j.annonc.2019.09.007 31912782 PMC8195110

[16] Fang X, Wei J, He X, et al. Q uantitative association between body mass index and the risk of cancer: a global Meta-analysis of prospective cohort studies. Int J Cancer. 2018;143(7):1595–603. 10.1002/ijc.31553 29696630

[17] Markozannes G, Tzoulaki I, Karli D, et al. Diet, body size, physical activity and risk of prostate cancer: an umbrella review of the evidence. Eur J Cancer. 2016;69:61–9. 10.1016/j.ejca.2016.09.026 27816833

[18] Harrison S, Tilling K, Turner EL, et al. Systematic review and meta-analysis of the associations between body mass index, prostate cancer, advanced prostate cancer, and prostate-specific antigen. Cancer Causes Contr. 2020;31(5):431–49. 10.1007/s10552-020-01291-3 PMC7105428 32162172

[19] Song M, Willett WC, Hu FB, et al. Trajectory of body shape across the lifespan and cancer risk. Int J Cancer. 2016;138(10):2383–95. 10.1002/ijc.29981 26704725 PMC5079685

[20] Wang Q-L, Song M, Clinton SK, et al. Longitudinal trajectories of lifetime body shape and prostate cancer angiogenesis. Eur J Epidemiol. 2022;37(3):261–70. 10.1007/s10654-021-00838-1 35025021

[21] Finley DS, Calvert VS, Inokuchi J, et al. Periprostatic adipose tissue as a modulator of prostate cancer aggressiveness. J Urol. 2009;182(4):1621–7. 10.1016/j.juro.2009.06.015 19683746

[22] Banez LL, Hamilton RJ, Partin AW, et al. Obesity-related plasma hemodilution and PSA concentration among men with prostate cancer. JAMA. 2007;298(19):2275–80. 10.1001/jama.298.19.2275 18029831

[23] Center MM, Jemal A, Lortet-Tieulent J, et al. International variation in prostate cancer incidence and mortality rates. Eur Urol. 2012;61(6):1079–92. 10.1016/j.eururo.2012.02.054 22424666

[24] Zhou CK, Check DP, Lortet-Tieulent J, et al. Prostate cancer incidence in 43 populations worldwide: an analysis of time trends overall and by age group. Int J Cancer. 2016;138(6):1388–400. 10.1002/ijc.29894 26488767 PMC4712103

[25] Wang K, Chen X, Gerke TA, Bird VY, Ghayee HK, Prosperi M. BMI trajectories and risk of overall and grade-specific prostate cancer: an observational cohort study among men seen for prostatic conditions. Cancer Med. 2018;7(10):5272–80. 10.1002/cam4.1747 30207080 PMC6198207

[26] Keum N, Greenwood DC, Lee DH, et al. Adult weight gain and adiposity-related cancers: a dose-response meta-analysis of prospective observational studies. J Natl Cancer Inst. 2015;107(2):djv088. 10.1093/jnci/djv088 25757865

[27] Jacobsen BK, Eggen AE, Mathiesen EB, Wilsgaard T, Njølstad I. Cohort profile: the Tromso Study. Int J Epidemiol. 2012;41(4):961–7. 10.1093/ije/dyr049 21422063 PMC3429870

[28] Eggen AE, Mathiesen EB, Wilsgaard T, Jacobsen BK, Njølstad I. The sixth survey of the Tromsø study (Tromsø 6) in 2007–08: collaborative research in the interface between clinical medicine and epidemiology: study objectives, design, data collection procedures, and attendance in a multipurpose population-based health survey. Scand J Public Health. 2013;41(1):65–80. 10.1177/1403494812469851 23341355

[29] Brækkan SK, Hald EM, Mathiesen EB, et al. Competing risk of atherosclerotic risk factors for arterial and venous thrombosis in a general population: the Tromsø study. Arterioscler Thromb Vasc Biol. 2012;32(2):487–91. 10.1161/ATVBAHA.111.237545 22075253

[30] Epstein JI, Egevad L, Amin MB, Delahunt B, Srigley JR, Humphrey PA. The 2014 International Society of Urological Pathology (ISUP) Consensus Conference on Gleason Grading of Prostatic Carcinoma: definition of grading patterns and proposal for a new grading system. Am J Surg Pathol. 2016;40(2):244–52. 10.1097/PAS.0000000000000530 26492179

[31] Sobin LH, Compton CC. TNM seventh edition: what’s new, what’s changed: communication from the International Union Against Cancer and the American Joint Committee on Cancer. Cancer. 2010;116(22):5336–9. 10.1002/cncr.25537 20665503

[32] D’Amico AV, Whittington R, Malkowicz SB, et al. Predicting prostate specific antigen outcome preoperatively in the prostate specific antigen era. J Urol. 2001;166(6):2185–8. 10.1016/S0022-5347(05)65531-0 11696732

[33] Stevens J, Truesdale KP, McClain JE, Cai J. The definition of weight maintenance. Int J Obes. 2006;30(3):391–9. 10.1038/sj.ijo.0803175 16302013

[34] Kvåle R, Myklebust T, Engholm G, Heinävaara S, Wist E, Møller B. Prostate and breast cancer in four Nordic countries: a comparison of incidence and mortality trends across countries and age groups 1975–2013. Int J Cancer. 2017;141(11):2228–42. 10.1002/ijc.30924 28795403

[35] Ferlay J, Colombet M, Soerjomataram I, et al. Cancer incidence and mortality patterns in Europe: estimates for 40 countries and 25 major cancers in 2018. Eur J Cancer. 2018;103:356–87. 10.1016/j.ejca.2018.07.005 30100160

[36] Kvåle R, Møller B, Angelsen A, et al. Regional trends in prostate cancer incidence, treatment with curative intent and mortality in Norway 1980–2007. Cancer Epidemiol. 2010;34(4):359–67. 10.1016/j.canep.2010.04.017 20627840

[37] Albertsen PC, Bjerner LJ, Pasovic L, et al. Opportunistic prostate-specific antigen testing in Norwegian men: a public health challenge. BJU Int. 2024;133(1):104–11. 10.1111/bju.16211 37869764 PMC10842188

[38] Discacciati A, Orsini N, Andersson S-O, Andrén O, Johansson J, Wolk A. Body mass index in early and middle-late adulthood and risk of localised, advanced and fatal prostate cancer: a population-based prospective study. Br J Cancer. 2011;105(7):1061–8. 10.1038/bjc.2011.319 21847119 PMC3185939

[39] Renehan AG, Tyson M, Egger M, Heller RF, Zwahlen M. Body-mass index and incidence of cancer: a systematic review and meta-analysis of prospective observational studies. Lancet. 2008;371(9612):569–78. 10.1016/S0140-6736(08)60269-X 18280327

[40] MacInnis RJ, English DR. Body size and composition and prostate cancer risk: systematic review and meta-regression analysis. Cancer Causes Contr. 2006;17(8):989–1003. 10.1007/s10552-006-0049-z 16933050

[41] Fang Z, Giovannucci EL. The timing of adiposity and changes in the life course on the risk of cancer. Cancer Metastasis Rev. 2022;41(3):471–89. 10.1007/s10555-022-10054-2 35908000

[42] Yuan C, Jian Z, Feng S, et al. Do obesity-related traits affect prostate cancer risk through serum testosterone? A Mendelian randomization study. Cancers. 2023;15(19):4884. 10.3390/cancers15194884 37835578 PMC10571835

[43] Ramalingam S, Ramamurthy VP, Njar VCO. Dissecting major signaling pathways in prostate cancer development and progression: mechanisms and novel therapeutic targets. J Steroid Biochem Mol Biol. 2017;166:16–27. 10.1016/j.jsbmb.2016.07.006 27481707 PMC7371258

[44] Williams G. Aromatase up-regulation, insulin and raised intracellular oestrogens in men, induce adiposity, metabolic syndrome and prostate disease, via aberrant ER-α and GPER signalling. Mol Cell Endocrinol. 2012;351(2):269–78. 10.1016/j.mce.2011.12.017 22233684

[45] Massillo C, Dalton GN, Porretti J, et al. CTBP1/CYP19A1/estradiol axis together with adipose tissue impacts over prostate cancer growth associated to metabolic syndrome. Int J Cancer. 2019;144(5): 1115–27. 10.1002/ijc.31773 30152543

[46] Omran A, Leca BM, Oštarijaš E, et al. Metabolic syndrome is associated with prostate enlargement: a systematic review, meta-analysis, and meta-regression on patients with lower urinary tract symptom factors. Ther Adv Endocrinol Metab. 2021;12:1–29 10.1177/20420188211066210 PMC8664322 34900218

[47] Chen W, Man S, Wang B, Kadeerhan G, Huang X. Metabolically healthy obesity is associated with increased risk of lower urinary tract symptoms secondary to benign prostatic hyperplasia: a cohort study of C hinese elderly males. LUTS: Low Urin Tract Symptoms. 2022;14(3):170–7. 10.1111/luts.12420 34882977

[48] Al-Zubaidi M, Hawks C, Fernando S, et al. Relationship between lower urinary tract symptoms (LUTS) and prostate cancer: a persistent myth. J Clin Urol. 2023:20514158231170420. 2023;0(0). 10.1177/20514158231170420

[49] Chandra Engel J, Palsdottir T, Aly M, et al. Lower urinary tract symptoms (LUTS) are not associated with an increased risk of prostate cancer in men 50–69 years with PSA≥ 3 ng/ml. Scand J Urol. 2020;54(1):1–6. 10.1080/21681805.2019.1703806 31876229

[50] Luo R, Chen Y, Ran K, Jiang Q. Effect of obesity on the prognosis and recurrence of prostate cancer after radical prostatectomy: a meta-analysis. Transl Androl Urol. 2020;9(6):2713. 10.21037/tau-20-1352 33457243 PMC7807337

[51] Wilsgaard T, Jacobsen BK, Mathiesen EB, Njølstad I. Weight loss and mortality: a gender-specific analysis of the Tromsø study. Gend Med. 2009;6(4):575–86. 10.1016/j.genm.2009.12.003 20114008

[52] Larsen IK, Småstuen M, Johannesen TB, et al. Data quality at the Cancer Registry of Norway: an overview of comparability, completeness, validity and timeliness. Eur J Cancer. 2009;45(7):1218–31. 10.1016/j.ejca.2008.10.037 19091545

