# Supplementary material for: Excess weight, weight gain, and prostate cancer risk and prognosis: the PROCA-life study
Source: Acta Oncol. 2024 Apr 9;63:32953. doi: 10.2340/1651-226X.2024.32953 (PMC11332472; doi:10.2340/1651-226X.2024.32953)

Supplementary material has been published as submitted. It has not been copyedited or typeset by Acta Oncologica.

Supplementary Figure.

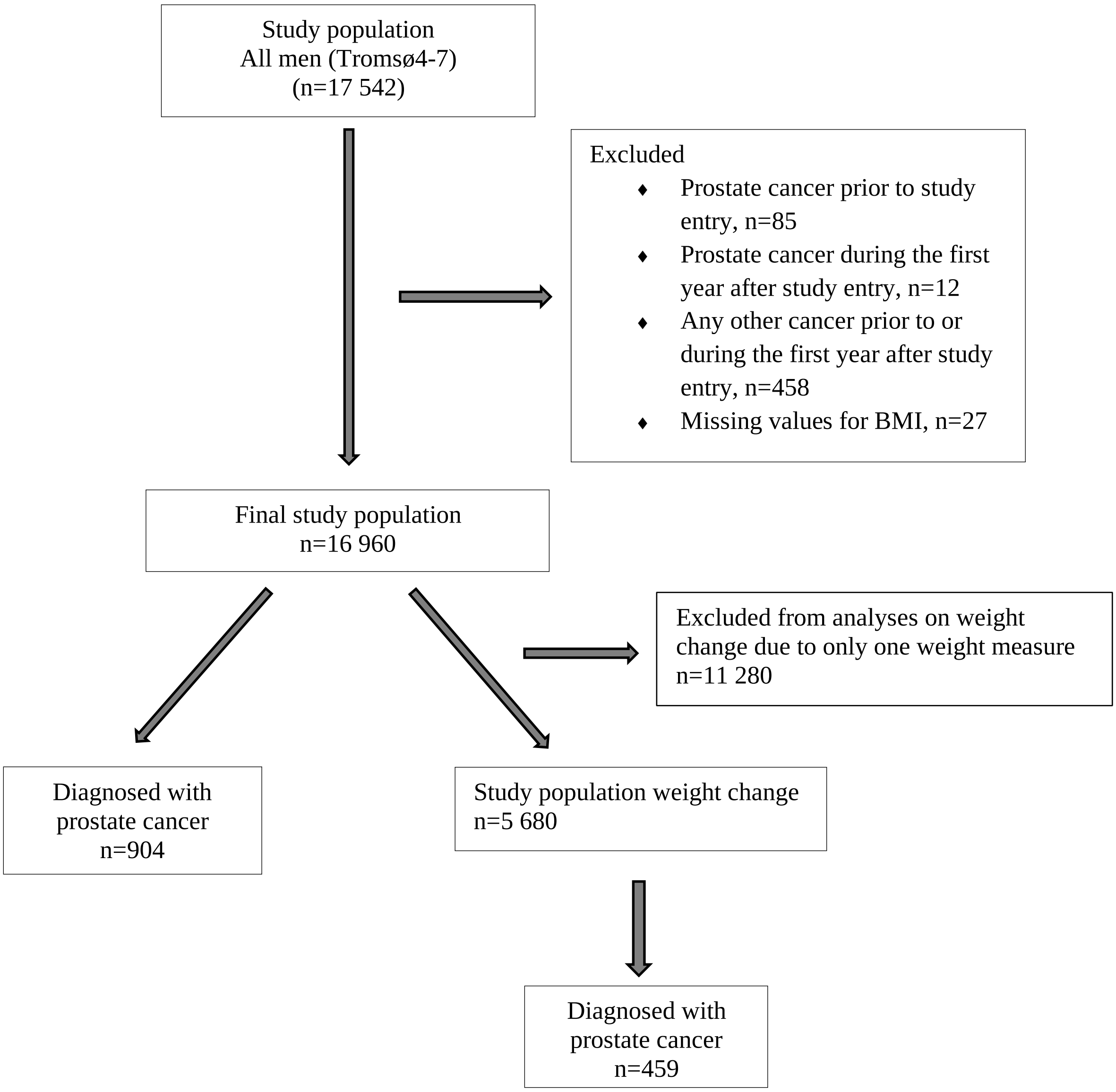

Supplement: Excess weight, weight gain, and prostate cancer risk and prognosis: the PROCA-life study [file AO-63-32953-s1.pdf]
