# Supplementary material for: Excess weight, weight gain, and prostate cancer risk and prognosis: the PROCA-life study
Source: Acta Oncol. 2024 Apr 9;63:32953. doi: 10.2340/1651-226X.2024.32953 (PMC11332472; doi:10.2340/1651-226X.2024.32953)
Supplement: Excess weight, weight gain, and prostate cancer risk and prognosis: the PROCA-life study [file AO-63-32953-s2.pdf]

Supplementary material has been published as submitted. It has not been copyedited or typeset by Acta Oncologica.

Supplementary table. Hazard ratios (HRs) for incident prostate cancer according to pre-diagnostic body mass index (BMI) stratified by age groups (tertiles) at entry. The PROCA *life* Study.

		<40 years of age			40-50 years of age			>50 years of age	
		**Model 1[a]**	**Model 2[b]**		**Model 1[a]**	**Model 2[b]**		**Model 1[a]**	**Model 2[b]**
		HR (95% CI)	HR (95% CI)		HR (95% CI)	HR (95% CI)		HR (95% CI)	HR (95% CI)
**BMI entry (kg/m^2)**	*N=*[c]			*N=*[c]			*N=*[c]		
<25.0	*28/2195*	*1 (Reference)*	*1 (Reference)*	*141/1799*	*1 (Reference)*	*1 (Reference)*	*196/1627*	*1 (Reference)*	*1 (Reference)*
25.0-<30	24/1509	1.15 (0.66-1.98)	1.14 (0.66-1.97)	158/1944	0.95 (0.76-1.20)	0.95 (0.76-1.20)	215/2095	0.80 (0.66-0.97)	0.79 (0.65-0.96)
≥ 30	3/282	0.83 (0.25-2.73)	0.85 (0.26-2.82)	35/432	0.99 (0.68-1.43)	0.99 (0.68-1.44)	48/476	0.86 (0.63-1.19)	0.85 (0.62-1.18)
P trend		0.911	0.903		0.793	0.815		0.082	0.068
**BMI Continuous**									
Per SD [d]		1.10 (0.81-1.49)	1.10 (0.81-1.50)		0.97 (0.86-1.10)	0.97 (0.85-1.09)		0.93 (0.84-1.04)	0.93 (0.83-1.04)

[a] Adjusted for age.
[b] Adjusted for smoking, physical activity, education level, and alcohol at the same time of baseline.
[c] Number of incident prostate cancer cases
[d] Standard deviation for BMI was calculated to 4 kg/m^2
